# Mitocanic Di- and Triterpenoid Rhodamine B Conjugates

**DOI:** 10.3390/molecules25225443

**Published:** 2020-11-20

**Authors:** Sophie Hoenke, Immo Serbian, Hans-Peter Deigner, René Csuk

**Affiliations:** 1 Organic Chemistry, Martin-Luther University Halle-Wittenberg, Kurt-Mothes Street 2, D-06120 Halle, Germany; sophie.hoenke@chemie.uni-halle.de (S.H.); immo.serbian@chemie.uni-halle.de (I.S.); 2 Medical and Life Science Faculty, Institute of Precision Medicine, Furtwangen University, Jakob–Kienzle–Street 17, D-78054 Villigen–Schwenningen, Germany; dei@hs-furtwangen.de

**Keywords:** triterpenoic acid, maslinic acid, tormentic acid, betulinic acid, oleanolic acid, rhodamine B, cytotoxicity

## Abstract

The combination of the “correct” triterpenoid, the “correct” spacer and rhodamine B (**RhoB**) seems to be decisive for the ability of the conjugate to accumulate in mitochondria. So far, several triterpenoid rhodamine B conjugates have been prepared and screened for their cytotoxic activity. To obtain cytotoxic compounds with EC_50_ values in a low nano-molar range combined with good tumor/non-tumor selectivity, the Rho B unit has to be attached via an amine spacer to the terpenoid skeleton. To avoid spirolactamization, secondary amines have to be used. First results indicate that a homopiperazinyl spacer is superior to a piperazinyl spacer. Hybrids derived from maslinic acid or tormentic acid are superior to those from oleanolic, ursolic, glycyrrhetinic or euscaphic acid. Thus, a tormentic acid-derived **RhoB** conjugate **32**, holding a homopiperazinyl spacer can be regarded, at present, as the most promising candidate for further biological studies.

## 1. Introduction

Cancer remains one of the leading causes of death worldwide, and the incidence is increasing. Cancer is the second-leading cause of death globally, accounting for 9.6 million deaths in 2018 [1]. It is expected that in 2030, 21 million people worldwide will suffer from cancer [2]. Tremendous progress, however, has been made in the treatment of individual cancers [3,4,5,6]. This is due, on one hand, to improved early detection and prophylaxis and, on the other, to the development of highly efficient drugs in a wide range of different substance classes. Thus, the probability of premature death from cancer per year decreased from 8.3% in the year 2000 to 6.9% in 2015. It is expected to be as low as 5.3% in 2030, saving approximately 1.1 million lives per year [7].

Both proteins and small molecules have proven their worth in therapy and are in use in the clinic. However, low-molecular-weight drugs often bear the stigma of reduced selectivity in which the cytotoxic drug not only targets cancerous cells, but it also damages healthy tissue. This increases serious side effects and stress symptoms in patients, including nausea, heart or brain damage, impairments to the central nervous system and damage to cells of the inner ear; losses of fertility, hearing and hair have also been noted [8].

These serious side effects and impairments limit the use and acceptance of a drug, as they reduce patient compliance due to a significantly reduced quality of life. This not only endangers the chances of successful therapy, but it often also leads to discontinuation of the therapy [9,10].

Since the problems of reconversion of cancer cells into normal cells (“reprogramming”, for example, of terminally differentiated cancer cells into cancer cells of benign phenotypes) [11] have only remotely been solved today, cancer cells have to be removed either by surgery or destroyed by radiation or chemotherapy. Cell death by chemotherapy can be induced in many different ways [12,13,14,15,16,17], but the mitochondria play a major role in the life or death of a cell. Thus, agents that target mitochondria and induce a controlled cell death, so called “mitocans”, have received increased attention in recent years [17,18,19,20,21,22,23,24,25,26,27,28,29,30,31,32,33,34,35,36,37,38]. This seems even more significant inasmuch as cancer cells are closely linked to dysregulated apoptosis of the cells; as a consequence, drug resistance of the cancer cells can develop [39].

Mitocans (as well as other cytotoxic agents) are often able to induce apoptosis; however, the death of a cell, irrespective of whether this cell is malignant, is not random at all. Triggering of controlled cell death is always preferable to an unselective rupture of membranes following the application of extreme but locally applied heat, freeze–thaw cycles or steep osmotic gradients. Controlled cell death can be triggered on a cellular level from nuclear, reticular, cytoskeletal, lysosomal, membrane or, most important, mitochondrial origins [16].

Usually, the cells in a living organism closely cooperate, and cells are constantly in an equilibrium between life and death. Triggering programmed cell death routines removes damaged, infected and out-of-control cells from the organism. The problem arises from the latter cells, since most cancer cells do not respond to extrinsic apoptotic triggers. Thus, mitochondria present a target of emerging interest for cancer therapy as they can trigger apoptosis through an intrinsic pathway. Apoptosis usually starts with loss of mitochondrial membrane potential, followed by the release of cytochrome c and activation of caspase 3 [40]. Furthermore, permeabilization of the outer mitochondrial membrane and the release of cytochrome c are required in many cell death stimuli [41]. This release of cytochrome c can be regarded as a “point of no return” finally leading to the death of the cell. This highlights the importance of mitochondrial compartmentation and explains the devastating effect following permeabilization of the mitochondrial membrane [42,43,44]. In addition to membrane permeabilization, the opening of the mitochondrial permeability transition pore is also considered an important event resulting in mitochondrial depolarization and the release of apoptotic factors [29].

In recent years, triterpenes have repeatedly and increasingly entered the focus of scientific interest. Extensive studies on their apoptotic and cytotoxic properties have been performed. A major concern in dealing with cancer is the MDR (multiple drug resistance) phenotype [39]. These cancer cells overexpress ATP-dependent transporters that eject toxic compounds from the cell before they cause harm to the cell. Some triterpenes are known inhibitors of the efflux pump MDR1, but they are also known to downregulate the transcription factor NF-κB. For cancer, it is widely accepted [45] that NF-κB promotes tumor migration and tumor proliferation.

## 2. Results

Mitochondrial membranes of malignant cells hold an increased membrane potential compared to non–malignant cells [46]. This effect fosters the accumulation of cationic molecules [17,47,48], hence inducing high selectivity for mitocans holding a (more or less) lipophilic cation such as a rhodamine scaffold. The same effect applies for triphenylphosphonium cations [49,50,51,52,53,54,55,56,57] and to a small extent for quaternary ammonium ions [58,59,60], zwitterionic *N*-oxides [60,61] and triterpenes substituted with BODIPYs [62,63,64,65,66] or a safirinium moiety [67]. However, the presence of a cationic center is not alone decisive for achieving high cytotoxic effects [60].

**Figure 1:**
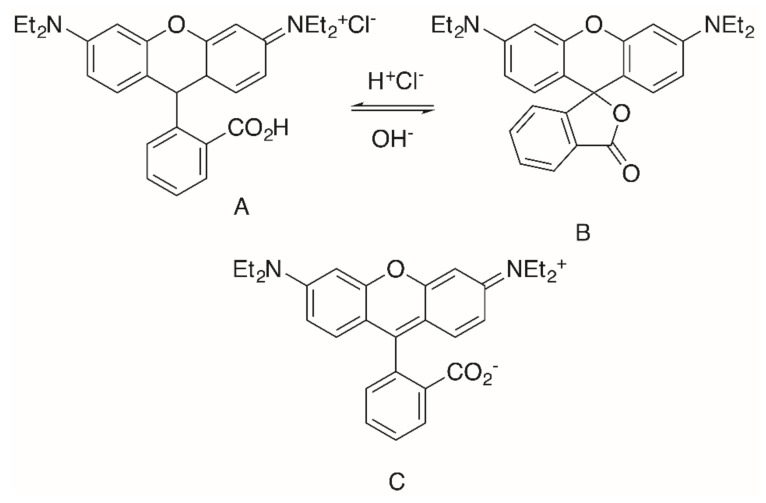
Structure of Rhodamine B (**Rho**B) in its “open” form **A**, “closed” lactone form **B** and the zwitterion **C**.

Rhodamine B (**RhoB**) seems to be a privileged scaffold. This fluorescent dye, also known as rhodamine 610, C.I. Pigment violet 1, basic violet 10, and C.I. 45170 [68], was invented in 1888 (“Tetraethyl-rhodamine”) by M. Cérésole [69,70], and since then it has been widely used in biology, biotechnology and as a biosensor [71,72]. **RhoB** exists in an equilibrium [73,74,75,76,77] between an “open” positively charged form A (Figure 1) that is fluorescent and a “closed”, non–fluorescent form B. Under acidic conditions, pink-colored A dominates, while colorless B dominates under basic conditions. Further, in less polar organic solvents, the zwitterionic form C undergoes a rapid reversible conversion to B [78,79,80,81].

**RhoB** is suspected to be carcinogenic [82,83,84,85]. The LD_50_ value for orally administered RhoB in rats is >500 mg/kg, and an older report classified **RhoB** (as well as Rho6G) as possibly carcinogenic in rats [85]. **RhoB**, however, seems not to be mutagenic in Chinese hamster ovary cells [86], but it presents a genotoxic hazard for mammalian organisms [87]. As far as the **RhoB**–triterpene conjugates are concerned, two types of compounds have been accessed so far: triterpenes with a **RhoB** moiety directly attached to the skeleton of the triterpene, and compounds wherein these two units are separated by a suitable spacer.

**Figure 2:**
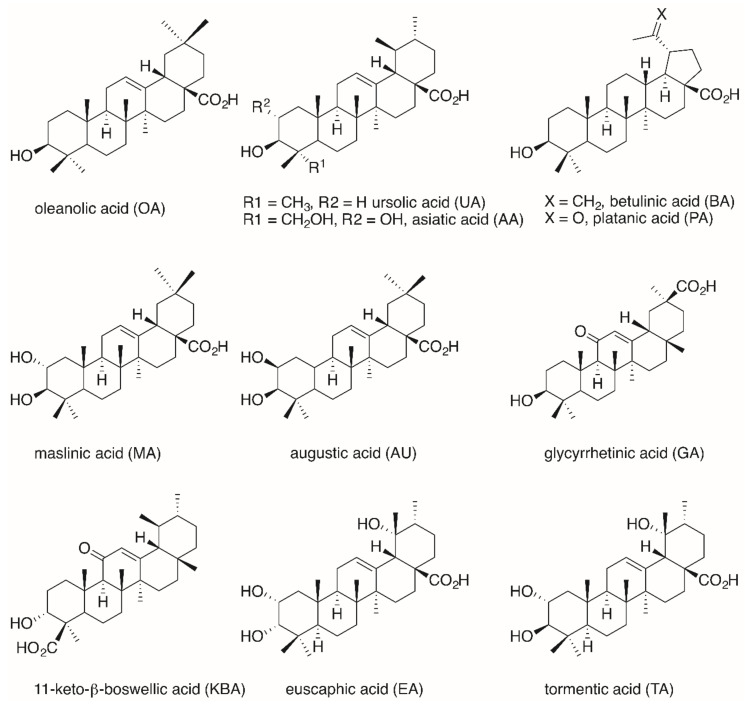
Structure of some important pentacyclic triterpenoic acids.

To date, hybrid molecules have been prepared from oleanolic acid (**OA**, Figure 2), ursolic acid (**UA**), glycyrrhetinic acid (**GA**), betulinic acid (**BA**), maslinic acid (**MA**), augustic acid (**AU**), 11-keto-β-boswellic acid (**KBA**), asiatic acid (**AA**), tormentic acid (**TA**) and euscaphic acid (**EA**).

By means of suitable double-staining experiments, it could be shown that these hybrids are actually effective as mitocans [88], and preliminary molecular modeling studies suggest these compounds might target the mitochondrial NADH dehydrogenase and mitochondrial succinate dehydrogenase [89]. Both enzymes are part of the mitochondrial electron transport chain; this also suggests an increased production of reactive oxygen species (ROS). An increased production of ROS would lead to an oxidative damage of the cell and trigger apoptosis through an intrinsic pathway. Therefore, the integrity of the **RhoB** basic structure seems to be of crucial importance. It has been shown that derivatives from the triphenylmethane dye malachite green still exhibit increased cytotoxicity as compared to their parent compounds [90]. The cytotoxicity, however, of these hybrids was much lower than those observed for the **RhoB** derivatives (vide infra).

**Figure 3:**
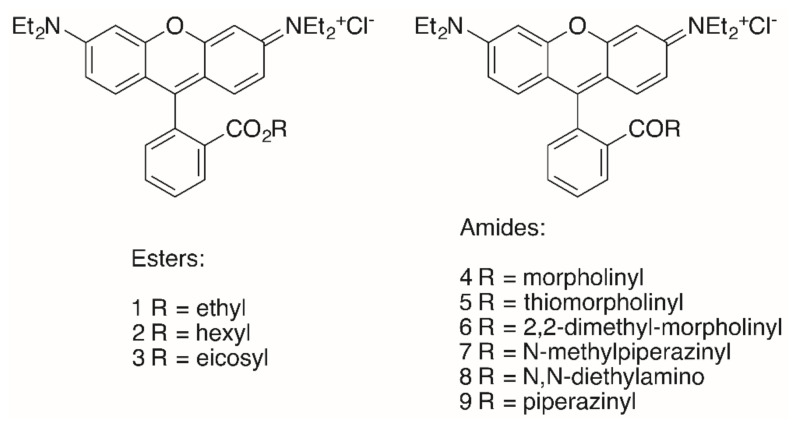
Structures of “simple” **RhoB** conjugates **1**–**9**.

The triterpenoid skeleton is equally important. Here, too, it was shown that “simple” RhoB conjugates **1**–**9** (Figure 3) also had lower cytotoxicity than the corresponding triterpenoid analogs, but their tumor cell/non-tumor cell selectivity was also diminished (Table 1) [91].

Of special interest seems the morpholinyl derivative **4** inasmuch as this compound held the highest selectivity of this series with respect to MCF-7 carcinoma cells (S = (EC_50, NIH 3T3_ / EC_50, MCF-7_) > 19.5) and A2780 ovarian cancer cells (S = (EC_50, NIH 3T3_ / EC_50, A27807_) > 18.1) [90].

The highest cytotoxicity was observed for the hexyl ester **2** (EC_50_ = 0.15–0.19 μM) for the different tumor cell lines. Interestingly, an eicosyl ester **3** with a lipophilicity similar to that of triterpenoids did not show even moderate cytotoxicity [90], while hydroxycinnamic acid rhodamine B conjugates displayed good cytotoxicity in the low μM range [92].

**Figure 4:**
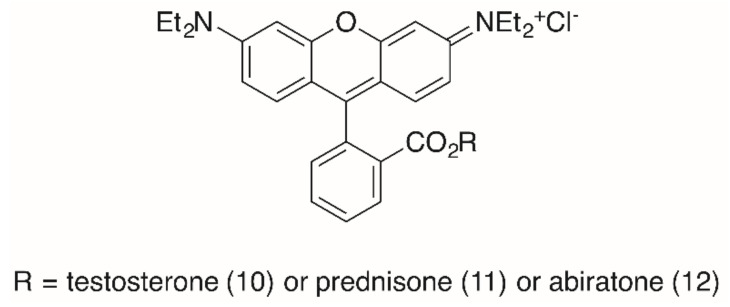
**RhoB** steroid conjugates from the esterification of RhoB with testosterone (→ **10**), prednisone (→ **11**) or abiratone (→ **12**), respectively.

The importance of the presence of a triterpenoid backbone is also evident from studies concerning RhoB steroid conjugates (Figure 4) [93]. In these studies, the reaction of the steroids cholesterol, testosterone, prednisone and abiraterone with an activated **RhoB** chloride furnished ester conjugates holding low EC_50_ values (SRB assays with several human tumor cell lines, Table 2). Thus, a testosterone conjugate **10** held EC_50_ = 60 nm for MCF-7 cells, but acted by necrosis (20%, A2780 cells). A prednisone conjugate **11** was less cytotoxic (0.2 μM for MCF-7 cells) but acted in A2780 cells mainly by apoptosis (48%) and late apoptosis (14%). In addition, this compound showed a higher selectivity for the A2780 tumor cells (S = 73) than for NIH 3T3 fibroblasts. For comparison, an abiratone conjugate **12** was less cytotoxic and also less selective [93].

A closer look at the cell cycle by FACS (with A2780 cells) showed a decrease of the G1 and G2/M peak with an increase of cells in the S phase. For cells treated with **11,** the S phase peak and the subG1/apoptosis peak increased significantly. However, for all compounds the selectivity between tumor cells and non-malignant fibroblasts NIH 3T3 was small and never exceeded 7.3 (**11**, for MCF-7 cells) [93].

**Figure 5:**
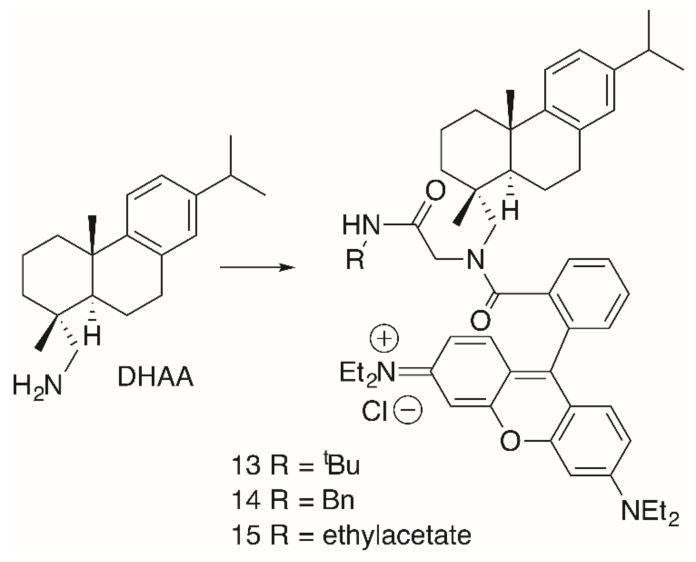
Dehydroabiethylamine (**DHAA**)-derived **RhoB** conjugates **13**–**15** obtained by Ugi-multi component reactions.

A similar behavior was observed for dehydroabietylamine (DHAA) derivatives **13**–**16** (Figure 5, Table 3). These products were easily obtained from dehydroabietylamine by the microwave-assisted multicomponent Ugi reaction using paraformaldehyde, an isocyanide and **RhoB** with yields between 47 and 50% [94].

**Figure 6:**
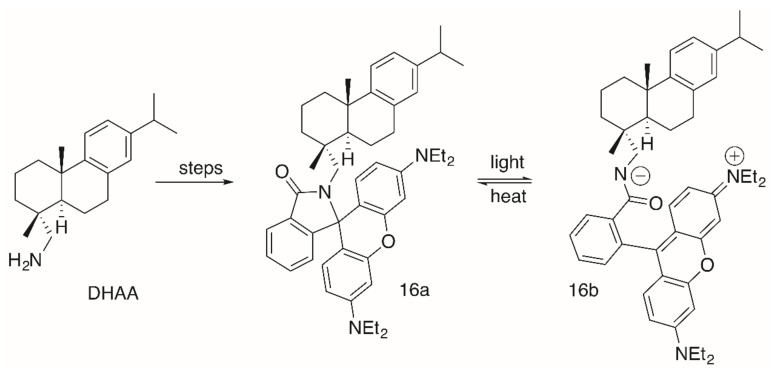
Synthesis of **DHAA**-derived **RhoB** conjugates **16a**/**16b** and their equilibrium.

Although the cytotoxicity of these compounds was good, their pharmacological potential was restricted by low selectivity values. Interestingly enough, products **16a**/**16b** (Figure 6), having been obtained from a simple Schotten–Baumann reaction with **DHAA** and **RhoB,** were not cytotoxic at all [94]. As mentioned above, **RhoB** conjugates derived from primary amines are able to form intramolecular non-fluorescent spirolactams (here **16a**). From a photo-induced ring opening reaction, **16b** was obtained from **16a** very quickly within 10 s of irradiation either with visible light or with UV light (λ = 254 or 366 nm). This equilibrium is also strongly influenced by changes in temperature, and at room temperature **16a** dominates the equilibrium [94].

**Figure 7:**
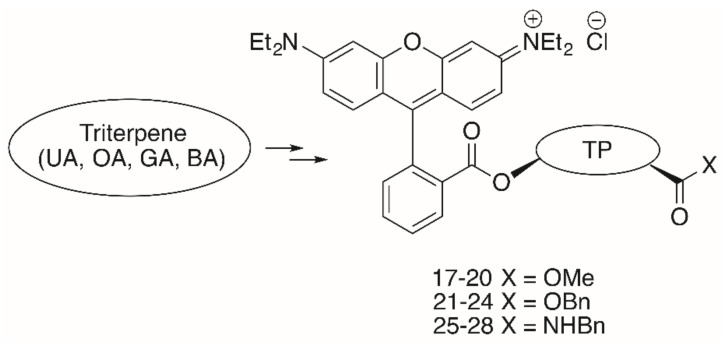
Un-spacered **UA-**, **OA-, GA-** and **BA**-derived esters **17**–**24** and amides **25**–**28**.

As far as the triterpene **RhoB** conjugates are concerned, the **RhoB** moiety can be attached to the triterpenoid scaffold either directly (e.g., in form of a triterpene **RhoB** ester) or with the aid of a suitable spacer. Pentacyclic triterpenoic acids (Figure 2) holding an **RhoB** moiety without an extra spacer have been prepared by esterification of **UA**, **OA**, **GA** and **BA** with **RhoB**, respectively, (Figure 7) [95].

All of these compounds had EC_50_ values between 0.02 and 15.8 μM (Table 4); thereby, the cytotoxicity of benzyl esters **21**–**24** was lower than the cytotoxicity of the methyl esters **17**–**20**, while the benzyl amides **25**–**28** were the most cytotoxic compounds of this series. The presence of a benzyl ester group as in **21**–**24** seems to be disadvantageous, while the opposite is true for the benzyl amides **25**–**28**. Compound **27** was the most cytotoxic compound (EC_50_ = 0.02–0.08 μM), but it was not selective for human tumor cells. Extra staining experiments showed this compound to be accumulated in the mitochondria of A2780 cells and to act mainly by apoptosis [95].

**Figure 8:**
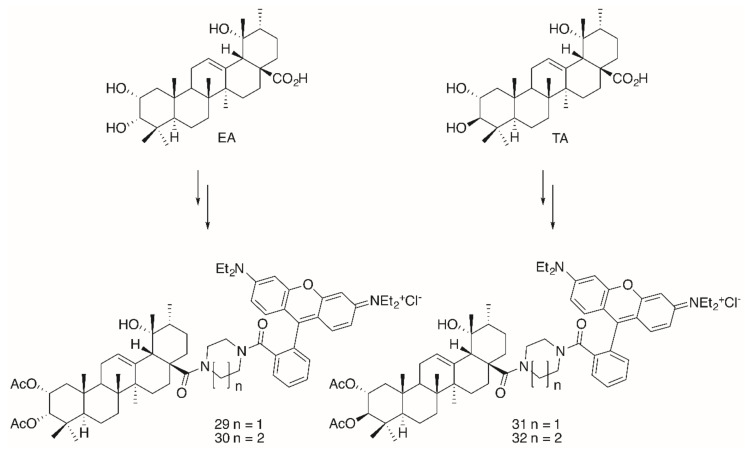
Synthesis of euscaphic (**EA**)- or tormentic acid (**TA**)-derived **RhoB** conjugates **29**–**32**.

Noteworthy in this context is the higher cytotoxicity of the glycyrrhetinic acid derivatives as compared to analogs derived from **OA**, **UA** or **BA**. Extensions in the design of these compounds led to the synthesis of triterpene conjugates with further modifications in the backbone (→ tormentic acid (**TA**) and euscaphic acid (**EA**)) as well as to changes in the ring size of the heterocyclic spacer between the backbone of the triterpene and the **RhoB** moiety (Figure 8).

**Figure 9:**
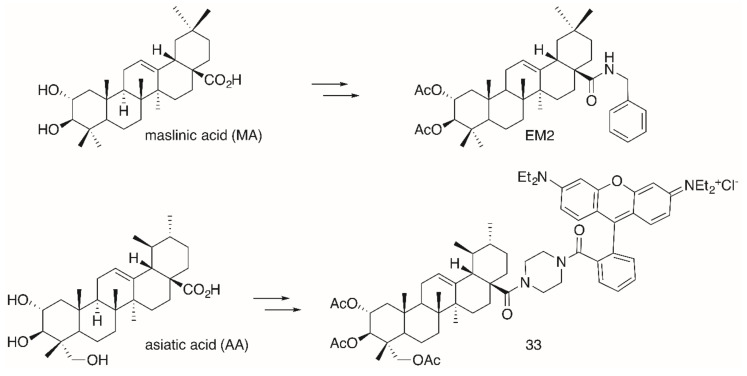
Synthesis of maslinic acid (**MA**)-derived **EM2** holding the same absolute configuration of hydroxyl groups in ring A as asiatic acid (**AA**)-derived conjugate **33**.

The significantly higher cytotoxicity (Table 5) of **TA-**derived **32** seems particularly noteworthy when comparing the different spacers: Thereby, the presence of a homopiperazinyl spacer [96] (as in **32**) proved to be clearly superior to the piperazinyl moiety (as in **31**). A similar trend was also noted for **EA-**derived compounds **29** and **30**. On the other hand, **TA-**derived compounds were more cytotoxic than the corresponding **EA** derivatives. Interestingly, the absolute configuration at C–2 and C–3 in **TA** corresponds exactly to the configuration found in maslinic acid (**MA**). Several **MA** derivatives (for example [97,98], a diacetylated benzylamide **EM2**, Figure 9) were of higher cytotoxicity and better selectivity than their corresponding **OA** or **UA** derivatives.

The same configuration is found in asiatic acid (**AA**). Again, its acetylated piperazinyl-rhodamine B conjugate **33** was most cytotoxic to many human tumor cell lines, being accumulated in the mitochondria, and it also acted as a mitocan [99]. However, for this compound an unusual non-linear rate of growth was detected for some human tumor cell lines (e.g., colorectal carcinoma HT29 and melanoma 518A2). In a bimodal manner at two different concentrations the tumor cells were killed, a phenomenon that might be due to an accelerated recovery of the mitochondrial membrane potential or due to a modulation of the mitochondrial permeability pores. However, at present a concentration-triggered activation of a metabolizing enzyme cannot completely be ruled out [99].

**Figure 10:**
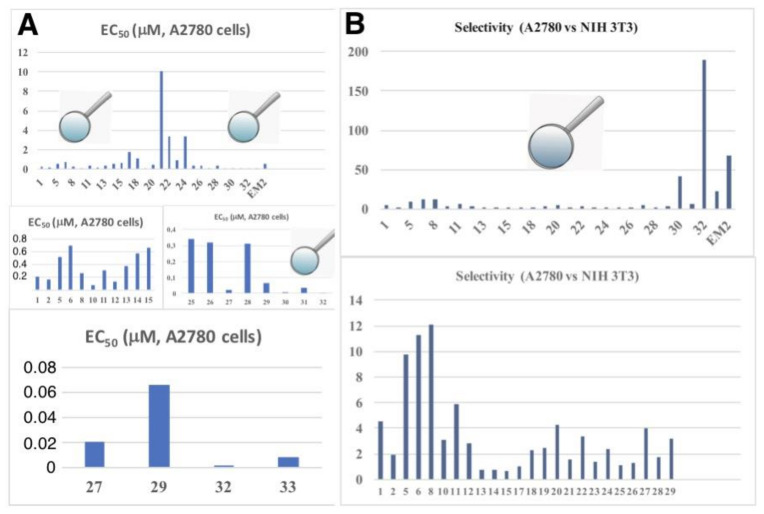
Graphical representation of the cytotoxicity of all compounds (EC_50_ in μM) for the cell line A2780 (**A**) combined with a comparison of tumor cell/non-tumor cell selectivity (A2780 vs. NIH 3T3, selectivity = (EC_50, NIH3T3_/EC_50, A2780_)) of all compounds (**B**).

A graphical comparison of all derivatives (using the target line A2780 as an example) is given in Figure 10 including a comparison of tumor cell/non-tumor cell selectivity (A2780 vs. NIH 3T3) of all compounds.

From Figure 10 the high potential of compound **32** (selectivity for A2780 or FaDu cells, ca. 190) becomes clearly visible, making this compound an interesting drug for advanced testing and biological screening.

## 3. Conclusions

**OA**-derived **RhoB** conjugates appear to be superior to analog **UA**-derived compounds in the majority of cases with respect to their cytotoxicity. Although **AKBA**-derived derivatives have good cytotoxicity properties, they were found to be less cytotoxic compared to other triterpene carboxylic acid derivatives, but they often showed better tumor cell/non-tumor cell selectivity. So far, the best cytotoxicity properties have been found for **MA-**, **EA-** and **TA-**derived derivatives. These allowed the transition to compounds of nano-molar activity, while many other triterpene carboxylic acid derivatives were cytotoxic only on a micro-molar concentration range. **MA-** derived derivatives seem to be approximately equivalent to **EA-**derived compounds. They are currently only surpassed in many tumor cell lines only by the analogous derivatives from **TA**. From results available so far, it can be concluded that compounds holding a homopiperazinyl spacer are superior to those with a piperazinyl spacer. This underlines the importance of the spacer for obtaining good cytotoxicity properties. Replacement of the secondary amide derived spacer by a primary amine like ethylenediamine has invariably led to **RhoB** conjugates of insignificant cytotoxicity (EC_50_ > 30 μM) due to the formation of a spirolactam holding no positive charge in the RhoB part.

However, the presence of a distal cation is not sufficient to obtain compounds with excellent cytotoxicity, as has been shown for several quaternary ammonium compounds or compounds where the **RhoB** part has been replaced by, for example, malachite green, a BODIPY residue or a safirinium group. In addition, the latter compounds do not act as mitocans, since their primary target is the endoplasmic reticulum.

A statement on the extent to which the replacement of the **RhoB** group with another rhodamine has a positive effect on biological activity cannot be made at present. The cytotoxic properties of these compounds, other spacers and other triterpene carboxylic acids are currently the subject of further investigation. The combination of the “correct” parent structure, the “correct” spacer and the “correct” **RhoB** seems to be decisive for the ability of the conjugate to accumulate in mitochondria. So far, a tormentic acid acid-derived **RhoB** conjugate **32** holding a homopiperazinyl spacer can be regarded as the most promising candidate for further biological studies. At present, no extended investigations have been carried out on the precise mode of action of these molecules.

## Figures and Tables

**Table 1:** Cytotoxicity of selected “simple” **RhoB** conjugates.

Compound	A375	HT29	MCF-7	A2780	FaDu	NIH 3T3
**RhoB**	>30	>30	>30	>30	>30	>30
**1**	0.38	0.41	0.23	0.21	0.30	0.96
**2**	0.19	0.19	0.14	0.17	0.15	0.32
**3**	>30	>30	>30	>30	>30	>30
**4**	7.09	5.46	1.54	1.66	4.53	>30
**5**	1.79	1.54	0.44	0.52	1.12	5.09
**6**	3.05	1.74	0.49	0.70	1.52	7.92
**7**	16.05	17.34	3.74	3.62	11.78	>30
**8**	1.03	0.54	0.32	0.27	0.64	3.27
**9**	>30	>30	17.80	26.40	>30	>30

EC_50_ in μM from SRB assays; cut-off 30 μM.

**Table 2:** Cytotoxicity of selected steroidal **RhoB** conjugates.

Compound	A375	HT29	MCF-7	A2780	FaDu	NIH 3T3
**10**	0.16	0.12	0.06	0.08	0.26	0.25
**11**	0.11	0.64	0.21	0.31	0.40	1.81
**12**	0.22	0.21	0.23	0.13	0.24	0.37

EC_50_ in μM from SRB assays.

**Table 3:** Cytotoxicity of selected **DHAA**-derived **RhoB** conjugates.

Compound	A375	HT29	MCF-7	A2780	FaDu	NIH 3T3
**13**	3.2	0.18	0.10	0.37	0.23	0.28
**14**	0.23	0.32	0.16	0.57	0.35	0.41
**15**	0.20	0.28	0.12	0.66	0.32	0.44
**16a/16b**	>30	>30	>30	>30	>30	>30

EC_50_ in μM from SRB assays.

**Table 4:** Cytotoxicity of un-spacered esters **UA-**, **OA-, GA-** and **BA**-derived esters **17**–**24** and amides **25**–**28**.

Compound	TP	R	FaDu	A2780	HT29	MCF-7	SW1736	NIH 3T3
**17**	UA	OMe	1.96	1.75	1.85	1.83	1.72	1.84
**18**	OA	OMe	1.99	1.14	2.75	2.31	1.76	2.63
**19**	GA	OMe	0.19	0.08	0.15	0.18	0.15	0.20
**20**	BA	OMe	1.29	0.42	0.61	0.81	0.74	1.77
**21**	UA	OBn	15.79	10.10	11.41	13.75	12.66	15.42
**22**	OA	OBn	9.12	3.35	8.90	9.40	9.05	11.25
**23**	GA	OBn	1.54	0.90	1.42	1.47	1.13	1.28
**24**	BA	OBn	7.59	3.36	5.33	5.05	6.43	8.04
**25**	UA	NBn	0.44	0.34	0.45	0.30	0.24	0.37
**26**	OA	NBn	0.50	0.32	0.46	0.36	0.27	0.40
**27**	GA	NBn	0.06	0.02	0.06	0.04	0.04	0.08
**28**	BA	NBn	0.54	0.31	0.53	0.47	0.45	0.54

EC_50_ in μM from SRB assays.

**Table 5:** Cytotoxicity of euscaphic (**EA**)- or tormentic acid (**TA**)-derived **RhoB** conjugates **29**–**32**, asiatic acid (**AA**)-derived **33** and maslinic acid (**MA**)-derived amide **EM2**.

Compound	A375	HT29	MCF-7	A2780	FaDu	NIH 3T3
**29**	0.19	0,19	0.094	0.066	0.074	0.21
**30**	0.012	0.012	0.022	0.004	0.004	0.164
**31**	0.14	0.16	0.0084	0.037	0.041	0.25
**32**	0.06	0.005	0.008	0.001	0.001	0.19
**33**	n.d.	0.017	0.012	0.008	n.d.	0.178
**EM2**	n.d.	4.70	7.70	0.50	n.d.	33.8

EC_50_ in μM from SRB assays.
